# The Productivity Dilemma in Workplace Health Promotion

**DOI:** 10.1155/2015/937063

**Published:** 2015-08-25

**Authors:** Martin Cherniack

**Affiliations:** Department of Medicine, University of Connecticut Health Center, 263 Farmington Avenue, Farmington, CT 06030-2017, USA

## Abstract

*Background*. Worksite-based programs to improve workforce health and well-being (Workplace Health Promotion (WHP)) have been advanced as conduits for improved worker productivity and decreased health care costs. There has been a countervailing health economics contention that return on investment (ROI) does not merit preventive health investment. *Methods/Procedures*. Pertinent studies were reviewed and results reconsidered. A simple economic model is presented based on conventional and alternate assumptions used in cost benefit analysis (CBA), such as discounting and negative value. The issues are presented in the format of 3 conceptual dilemmas. *Principal Findings*. In some occupations such as nursing, the utility of patient survival and staff health is undervalued. WHP may miss important components of work related health risk. Altering assumptions on discounting and eliminating the drag of negative value radically change the CBA value. *Significance*. Simple monetization of a work life and calculation of return on workforce health investment as a simple alternate opportunity involve highly selective interpretations of productivity and utility.

## 1. Introduction

The emphasis on workforce health and well-being as a component of a corporation's value has inspired a series of novel terms, such as presenteeism and salutogenesis, that are descriptive of Workplace Health Promotion (WHP) [1, 2]. Because of the central role of companies in financing health care in the United States, WHP has assumed the particular formulation of a conduit to reduce health care costs. In both Europe and North America, a common denominator for workplace health programs has been the potential increase of workplace productivity, hence the attention to* return on investment (ROI)* in WHP [3]. For purposes of clarification, the following represent two conventional ways of accounting for ROI, as either a* benefit cost ratio (BCR)* or* as net present value *(NPV):

 ROI: benefit/cost ratio, where discounted inflation-adjusted benefits are divided by costs.
 ROI: net present value/present value (NPV/PV of costs), where NPV is defined as the difference between the total discounted inflation-adjusted benefits and the costs of the program over its useful life.

In the United States, there has been a growing countervailing argument that capital investments in the health of the workforce do not afford appreciable monetized benefit in the short or intermediate term [4]. Hence, WHP is an imprudent commitment of resources, even when there are measurable health benefits [5]. More than a decade ago, Riedel et al. [6] observed the limited impact of improved health parameters on health care costs and predicted a greater emphasis on the productivity consequences of improved workforce health.

The effort to monetize health related performance effects has utilized a variety of approaches: (1) direct conversion, based on salary optimization, (2) introspective or self-descriptive surveys, and (3) firm-level or managerial evaluation. Collectively, these methods presuppose that impairment can be expensed as a proportion of annualized salary and that necessary refinements would include more accurate assessment of fringe benefits or localizing of labor replacement costs [5]. However, the problem is more involved than correcting for double counting or valuing service, education, or health care activities in a market economy. Large nonindustrial employers often have the capacity to negotiate group health directly by using self-insurance and scale with effects on health care costs that supersede individualized activity. They also can rapidly introduce or retract programmatic changes without jeopardizing operating budgets and can absorb effects on morale from reorganization of the workforce. Moreover, the optimistic assumption that WHP advantages will accrue to all parties (the company at the organizational level, the production unit, and the productive workforce) poses a series of potential dilemmas.

## 2. Methods/Procedures

The following analysis is posed in the form of three problems or dilemmas:

 Dilemma #1: the Productivity Paradox.
 Dilemma #2: the Problems of Accounting for Discounting and Negative Value.
 Dilemma #3: Monetizing the Multiple Dimensions of WHP, Value of a Working Life and a Saved Life.

The* Productivity Paradox* is treated as a nonstructured review and reconsideration of several seminal studies.* Dilemma #2* is an exercise in benefit cost analysis that substitutes alternate value for the discount rate and the determination of negative value.* Dilemma #3* is treated by introducing the economic concept of the* Value of a Statistical Life (VSL)* and comparing relative determinations.

## 3. Principal Findings

### 3.1. Dilemma #1: The Productivity Paradox

The* Productivity Paradox* is reflected in the well-recognized observation that increased productivity may generate* decreased* utility, and vice versa. The potential discordance between productivity and utility is appreciated in the traditional example of motor vehicle safety technology and traffic rules. Motor vehicle accidents are a productivity stimulator, generating economic activities in medical care, insurance, and vehicle repair and replacement. Safety technology adds costs and may diminish economic activity, but the argument for the utility of saved lives and reduced mortality has prevailed. The* Productivity Paradox* is influenced by cost benefit uncertainty, when the future value of expected benefits ≠ the present value of expected costs, and by the concept of Sunk Benefits, an intervention being discouraged by the negative opportunity cost of an unrecoverable fronted investment. In preventive health, at least in the United States, the determination is often weighted towards reducing sunk costs rather than towards nonevaluated health benefits [7].

**Figure 1:**
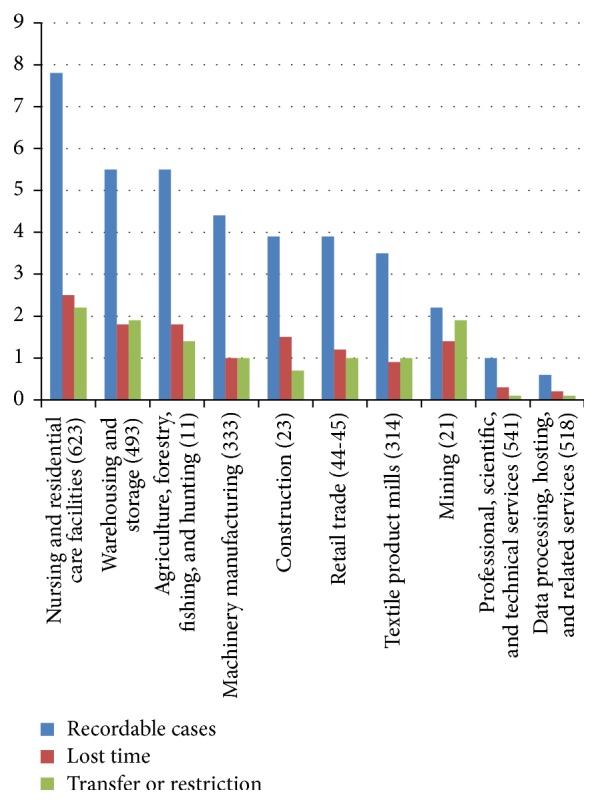
Work related injury by NAICS Sector, 2012, incidences per 1000.

Contradictory and even “nonrational” choices over valuing benefits and losses can be appreciated in an example from nursing in the United States. There is substantial evidence for compromised health in nurses, attributable to understaffing, shiftwork, work-family conflict, obesity, and depression [8–10].  Figure 1 presents recent North American Industrial Classification System (NAICS) data on injury and lost time rates for several occupational groups. The data confirms that nursing carries the greatest likelihood of injury related absenteeism of any industrial sector.  Table 1 summarizes results from two frequently cited studies on nurse understaffing and impacts on both patient mortality and nursing morale [11, 12]. The effect of understaffing on patient survival is apparently dire, with understaffing contributing as much as a 15% increase in patient mortality within 5 days of an understaffed shift. There have been significant and growing investments in capital intensive hospital based resources [13]. However, the 2011 IOM report entitled* The Future of Nursing*:* Leading Change, Advancing Health* indicates that the underinvestment in nursing has resulted in deficient education and training and insufficient numbers of personnel [14].

**Figure 2:**
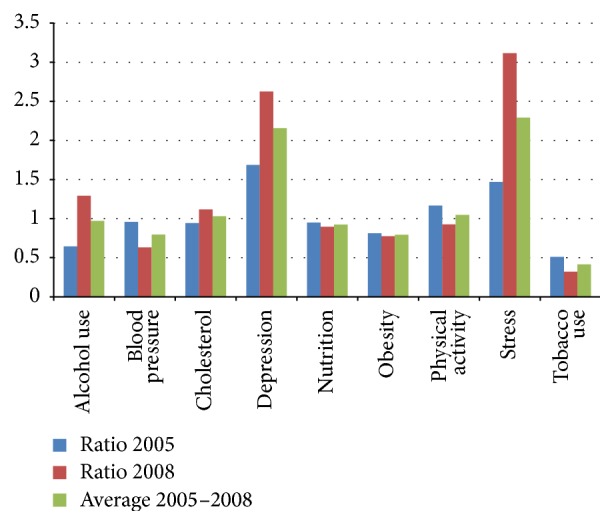
Johnson and Johnson Live for Life comparative risks per 100.

Figure 2 provides a different example of contradiction between productivity and health in terms of the differing implications of productivity improvements for the firm and the workforce. The Johnson and Johnson Company (J&J)* Live for Life Program* was inaugurated in 1979 and has been recognized and commercially disseminated as an effective Workplace Health Promotion (WHP) approach with its combination of health coaching, incentives, and onsite facilities [15]. The CEO of J&J promoted the interval from 2004 to 2008 as a period of high productivity due to genomics, information technology, and greater output ratios from the workforce. A goal of 9% productivity improvement per year was targeted. Over the same time period, J&J was recording an 11–15% annual increase in its yearly dividend (http://seekingalpha.com/article/1404151-a-new-normal-for-johnson-johnsons-dividend) without numerical expansion in its workforce (http://money.cnn.com/magazines/fortune/global500/2010/index.html.). Henke et al. [15] praised the effectiveness of the WHP activities of the company, in a comparison to other New Jersey companies. Figure 2 presents a reconsideration of their data showing the relative level of change in risk profiles. A comparison with other companies shows effective smoking cessation, mild improvement in blood pressure control and exercise, and no change in nutrition or obesity. There were also modest reductions in health costs over the 2004–2008 interval. There was, however, a dramatic increase in reported alcohol use, stress, and depression. Figure 2 is an interval comparison and reflects relative change detached from prevalence. While there is the likelihood of reduced attribution to overall population health from less commonly reported conditions (depression versus nutrition), the trend is, nevertheless, clear and ominous. Whether or not J&J's improved productivity has the consequence of deteriorating quality of life in its workforce cannot be inferred from the available data. It does suggest, however, that a much praised WHP program may have failed to address an ominous underside.

While an explanation of adverse health effects must be speculative, it is notable that the J&J program was a classic lifestyle-directed health promotion that included fitness centers and pedometry programs, healthier foods in cafeterias, Weight Watchers and other weight loss programs, and health coaching for blood pressure management, blood lipid control, smoking cessation, and chronic disease management. What was absent was inclusion of work conditions as predictive of health. The concept of integrating working conditions and work related well-being with more individualized WHP is the core concept in the NIOSH Total Worker Health Program (http://www.cdc.gov/niosh/twh/) [16, 17]. Our own health intervention work with corrections officers may also explain the J&J paradox. Targeted weight loss programs were highly effective [18], but in the setting of dramatically increased overtime demands, due to staffing levels, there was decreased sleep and increased depression, and stress, hypertension, and binge drinking all increased [19].

### 3.2. Dilemma #2: The Problems of Accounting for Discounting and Negative Value

Traditional health economics places an emphasis on discounting, the devaluing of future health benefits against the quantity of present investment. There are also assumptions of time preference that the future will be richer and more technologically proficient than the present, posing an argument for the lower relative cost of deferred investment. A third assumption involves risk avoidance, the bias against current investment by assuming deferred risk. Changing standard assumptions provides a different picture of the cost of nonintervention and delaying prevention.

One feature of the ROI formulae as presented in Section 1 is their presumption that the health and functional status of the workforce should be discounted to adjust for the decreased value of expected future benefits, given the benefit of an anticipated rate of return on an alternative investment. To clarify, discounting is a controversial but generally used mathematical calculation, which reduces future costs to a present value based on the assumption that an alternative rate of return on the intervention cost, such as the bond rate, was eschewed. The following conventional formula was obtained: 1/(1 + *r*)^*n*^, where *r* = discount rate and *n* = years into the future. An illustrative example was provided by Torgerson and Raftery on the cost and benefits of preventing hip fractures in older women [20]. The 10-year cost of daily supplemental calcium and vitamin D for 100 women is approximately $120,000. Without treatment, 16 hip fractures are predicted, but the regimen is expected to lower the incidence rate by 30%. The cost of preventing those 4.8 cases over 10 years would be $25,000 per prevented fracture, if no discounting was used. However, if a discount rate of 6% is adopted, the decision not to defer the cost of the intervention lowers its value by 56%. This is equivalent to a larger presumptive upfront cost of about $45,000 per prevented case. The presumption is that the cost of the intervention program is underestimated without discounting because an alternate return on investment was not accrued. Wherever a practical and ethical balance exists regarding the discounting, a large discount rate discourages a substantive investment in interventions by an employer, due to a large concealed cost. A second problem involves the accounting for negative value. Examples of negative value particularly pertinent to workforce health would include “intangibles” such as participatory time on a health and safety committee or cost and time invested in compliance with personal health surveillance protocols. For example, in the evaluation by Gowrisankaran et al. [4] of a WHP program for a hospital workforce, 82% participated; admissions for targeted conditions fell by 41%; and hospital admissions decreased overall by 12%. There was, however, a modest increase in health maintenance and primary care visits, which were assigned a negative value: a desired health outcome but a cost to the employer, and, in the authors' view, failure to overcome the hospital's hurdle rate.

Table 2 is a simplified presentation meant to demonstrate the limitations of and possible alternatives to conventional ROI calculation. In this model, the term* thin tailed health uncertainty* means that morbidity and mortality will be predictable and there is no need to provide for a catastrophic event effecting workforce health, such as an industrial explosion in a fertilizer plant, a consuming fire in a textile plant, or the likelihood that phthalates will induce male sterility.* No time preference* reflects the short duration of the interventions and precludes the expectation that American industrial society will necessarily be wealthier and more equitable in the near future and that future utility should be discounted in the present.* Hospitalization/WC* is an omnibus term representing health care costs that may be avoidable. These include reducing hospitalization or lost time from suboptimally managed chronic disease and reducing worker's compensation indemnity and medical costs due to a vigilant safety culture and more effective treatment and disability reduction. Three negative values are conceptualized:* quality of work life (QWL) activity*,* outpatient coaching (OPC), and workforce participation (WP).* QWL refers to workplace adaptation to workforce needs and includes mediations such as structured breaks, flexibility in work hours, and labor management relations that feature cooperation and participation. OPC is a preventive health concept that includes outpatient consultation or health coaching which are expected to improve general health or prevent the progression of morbidity where disease is established. WP is a compound outcomes variable that covers global engagement in health and participatory activities that can be separated from specific measured outcomes. These types of measures are used in the contemporary workplace. Quality of work life (QWL) can be assessed by survey and direct measure [21, 22]. Outpatient coaching has taken the form of direct contact and video and electronic formats. These include case review of chronic disease management compliance and motivational interviewing [23, 24]. Workforce participation can be assessed by engagement in and completion of specific program [25, 26] and also by direct participation in health related policy and program development at the level of the firm [27].

Negative value applies to related interventions that may be either a programmatic endpoint or a mediator of improved health and human performance. From the perspective of WHP, assigning negative value is an acknowledgement that engagement in some level of quality of life activity, such as greater physical activity, preservation of functional health, and involvement in a variety of work activities that add to the capacity of the workforce all have value. While negative value is customarily added to cost or subtracted from benefit, the converse does not seem to apply. The absence of these interventions does not alter the benefit cost ratio. The abbreviation EDU (equivalent dollar unit), the economic unit utilized in Table 2, is meant to underline that the examples are not meant to replicate real costs. Moreover, if the monetization of quality of work life is translational and a negative value is customarily associated with its implementation, an abstract unit of cost indicator seems the appropriate tender for quantifying work life into a rational economic choice.

While improved health and improved safety do not necessarily translate into reduced premium costs in the health care market, in this abstracted marketplace, the assumption is that improved health and performance does produce cost reduction and/or gain in income. Programmatic costs are self-explanatory and presume that program introduction occurs at the level of the company or employer. In fact, the evolution of the Accountable Care Organization (ACO) and medical home environments in the United States may lead to the transfer of some preventive functions to the healthcare service entity without cost implications for the employee. ACOs and medical homes are terms used to describe new integrated care models in the American health care system. Negative values have been explained, but their direct and indirect utility are central to Table 2.

The results show radically different outcomes for ROI based on the assumed value of the inputs. The principal benefits are health and performance related: reduced absenteeism and reduced group health and worker's compensation costs. If a conventional benefit cost ratio were calculated, and participation, health coaching, and QWL measures were treated as a cost, then the ROI of 0.70 would obviate workplace health related investment. Eliminating the discount rate, as has been suggested for climate change prevention [28], would appear to endorse a parallel presumption that health improvement at critical stages of life is less predictably linear than an alternative investment in, for example, a treasury bond. However, it would not alter the evident lack of positive return on investment.

The issues that are raised by neutralizing negative values or even accepting a positive value in the case of OPC require some elaboration. Participation rates, for example, do not necessarily require a time offset, any more than an 8-hour day represents (or once represented) a cost due to a circumscribed work week. Similarly, team meetings for managers are not customarily valued as a deferment from work responsibilities requiring a ROI calculation. In a related vein, acclimatizing an injured or impaired member of the workforce to a revised work pattern becomes an extravagant cost, but not when disability management is either a culturally accepted or regulated activity, as is the case for safety. A similar set of assumptions applies to QWL activity. If QWL activities are accepted as essential to workplace integrity in the same way that an HR department, a marketing group, or an IT department is considered implicitly necessary, then investment decisions are no longer absolute. Instead, funding questions are operational: internal provision versus outside vendor, scheduling and facilitation, or assignment of personnel. This evolution has already occurred in several hazardous duty workforces, such as police, fire, and corrections, where quality of life activities are essential components of the budget and organization. In Table 2, when negative value is simply removed from the BCR calculation, the ROI becomes favorable.

A final consideration involves reevaluating the OPC and treating it as a reduced cost, rather than as an expense (negative value) that may lead to reduced health care costs or higher output (productivity). The revision does not require an integration of a measure of productivity. Instead the presumption is that an effective OPC program would be encouraged by revisions in health care financing policy. For example, if the proposed premium reductions of the American health reform initiative, the Affordable Care Act (ACA), were directed to the firm that institutes an aggressive OPC program, rather than to the compliant individual, the result would be the equivalent of a credit. Similarly, if the services were offered by the ACO or medical home, provided there was active compliance on workplace access and with health and rehabilitation recommendations, then OPC would be either revenue neutral or could be structured as a discount. An additional idea that has been floated in economic circles is tax reduction based on domestic workforce investment, such as job creation, domestic purchasing, and healthy workplace programs [29]. While such measures do require considerable imagination and social optimism, they offer a pathway through incentivization to translate negative costs into a positive balance.

The recent experience in the United States with the Affordable Care Act (ACA) demonstrates complexities of incentivization, particularly when economic advantage or penalty is translated to the level of the individual [30, 31]. The ACA maintains essential American anomalies around health insurance—the provision of insurance plans through the employer and the predominant role of the private insurance industry in financing health plans. Workplace Health Promotion is an essential part of the legislation; however, its execution is fashioned around premium reductions for wellness compliant employees and, conversely, penalties for noncompliant plan participants. The ensuing controversies of rights of the disabled and economic penalties, as well as the failure to fund the accompanying policy research activities, have forestalled full implementation.

### 3.3. Dilemma #3: Monetizing the Multiple Dimensions of WHP, Value of a Working Life and a Saved Life

Cost-effectiveness ratios may be relevant for evaluating interventions in terms of health benefits, but ROI or monetary based valuations are putatively important for financial decision makers in the private sector, responsible for implementing WHP intervention and allocating necessary resources. To be computed accurately, such ROI calculation would require prospective studies that would measure cost and particularly benefits of WHP accurately from each relevant stakeholder's perspective. Bringing about an alignment of incentives to overcome barriers to implementation of WHP interventions also needs to account for differences in perspectives on value, and these are often masked by monetization. As an example, the replacement cost of loss of occupation due to morbidity will usually be a low multiple of wage or salary for an employer, but a disability adjusted life year (DALY) involves a considerably higher calculation for worker economic loss, leaving aside the more difficult issue of valuing an encumbered existence [32, 33].

In Table 3, the differing valuations of lost life and the economic consequences of disease are presented in the context of the value of a statistical life (VSL). The table is deliberatively misleading because WHP is a preventive policy with broad implementation and a critical effect on a population fraction; lost life or lost quality of life is a specific consequence. Nevertheless, the point of the table is to demonstrate scale. Health and safety interventions directed to the workforce are valued as a discriminant choice among other investment opportunities and with a narrow perspective on benefit. Moreover, the United States Environmental Protection Agency (EPA) valuation of a lost life or the estimation of a lost working life is not attached to a field of varying benefits; their magnitude is ultimately qualitative, an acknowledgement that human lives have a high value that can only be indirectly monetized.

## 4. Discussion

The discount rate depends on the assumption that the marginal efficiency of capital is greater than zero and the value of workforce health must exceed the anticipated rate of return on accumulated wealth. However, critical health events and biological aging are nonlinear; include concentrated, even catastrophic, changes in status and function; and instantaneously change from a high marginal cost rate to the extinction of all benefit. From a health perspective, preventing or limiting mortality or significant morbidity, if sufficiently valued, would merit an increase in the utility of interventions. If large enough, expected marginal utility to protect a working life becomes infinite. But this is, of course, an absurd supposition that either direct or socially transferred investment could ever approach 100%, if an absolute premium was placed on quality of life or survival. However, casual acceptance that workforce health promoting activities always represent a negative value is also an absurd supposition. To illustrate the point that the critical investment in diabetes prevention before clinical disease is present and adaptive physiological systems are exhausted does not represent an actual temporal choice, given that disease evolution involves critical and irreversible stages [34, 35]. Similarly, the investment in child care resources of nurses working 12-hour shifts has qualitative and quantitative consequences that can be monetized but has more complicated effects of discouraging nursing employment due to work family conflict [36].

It should be noted that in these examples the trade-off between risk aversion and so-called moral hazard is dismissed. The presumption is that a stable well-paid workforce will place implicit value on prevention of injury at work and on mitigating serious illness. More to the point, it is unclear that a meaningful trade-off is even appreciable since institutionalized decisions over health and preventive workplace interventions do not presume an individual assumption of risk. Programs that are introduced at the company or plan level are not necessarily subject to differential trade-offs, particularly when preventive interventions are not costly and not particularly differentiable. For example, the compliance functions of a health and safety committee or the public health interventions required of an effective vaccination program are not contingent on the graded assessment of risk. An individual decision not to vaccinate may be foolish or libertarian but it becomes relevant to public health investment if there is a socialization of risk of disease. The combination of regulation, usual safety practice, and public health professionalism is not commonly reduced to a menu of prices.

The appreciation of hazard on the part of employers and workers is often quite different. Several observers have noted an interesting anomaly in the economic literature; it is that while safety and cost are considered from a managerial perspective, the worker's own perceptions of risk are rarely included [37, 38]. Employers tend to put the weight of productivity loss on worker's lifestyle and health status, with particular weight on chronic diseases, lifestyle choices, and absenteeism [http://www.towerswatson.com/en-US/Insights/IC-Types/Survey-Research-Results/2013/09/2013-2014-stayingatwork-us-executive-summary-report]. Workers tend to attribute health and work problems to workload, problems of work-family balance, understaffing, poor supervisory relationships, and replacement by technology and job insecurity. Because both perspectives have legitimacy, the importance of workforce participation in decision making is functionally important. As Table 2 demonstrates, the application of discounting, the socialization of health care costs, and the translation of health effects as replacement costs all tend to deemphasize quality of work life, which is primarily the perspective of the employee.

In summary, the adjustments that are made to value the efficacy of health interventions at the workplace follow an idiosyncratic set of assumptions that limit consideration of quality of life and work. Facile assumptions around discounting and negative value place a grand hurdle before preventive interventions.

## Figures and Tables

**Table 1:** Consequences of nursing understaffing.

References		Outcome
Needleman et al., 2011 [11] (*n* = 197,961 admissions)	Shift with RN staffing level 8 hr or more below target during the first 5 days after admission (no patient time in ICU)	12% increase in patient mortality
	Shift with high patient turnover during the first 5 days after admission (no patient time in ICU)	12% increase in patient mortality
	Shift with high patient turnover during the first 5 days after admission (all admissions)	7% increase in patient mortality
	Shift with high patient turnover during the first 30 days after admission (all admissions)	4% increase in patient mortality

Aiken et al., 2002 [12] (*n* = 232,342 admissions)	Increase in 1 patient/RN/shift	(i) 7% increase in patient deaths within 30 days of admission (ii) 7% increase in failure to rescue (iii) 23% increase in RN burnout (iv) 15% increase in RN job satisfaction

**(a):** 

Assumptions		
	Duration	5 years
	Inflation rate	0%
	Discount rate per annum	4%
	Thin tailed health uncertainty	
	Time preference	None
	Risk aversion	N/A

Benefits		Cost

	Decreased absenteeism	75 EDU
	Decreased hospitalization/workers compensation	75 EDU

Costs		

	Programmatics	50 EDU
	Staffing	50 EDU

Negative value		

	QWL activity	25 EDU
	Increased OPC	25 EDU
	Workforce participation	25 EDU

EDU: equivalent dollar units.

**(b):** 

Outcomes		
	BCR^*∗*^	0.70
	BCR (no discount)	0.83
	No negative value (adjusted)	1.50
	OPC as a positive value	1.75

^*∗*^BCR: benefit cost ratio.

**Table 3:** The value of a life.

Period	Description	US $ value	Source
Annual or per event	Average investment in WHP	$144	Baker et al. 2008 [5]
	Occupational value of a statistical life year	$1,700.000	Moore and Viscusi 1990 [39]
	Implicit value of a statistical injury	$155,453 (nonsmoker-seat belt users) $83,186 (smoker-non-seat belt users)	Hersch and Pickton 1995 [40]
	Quality of life, one year (medical expenditure)	$129,000–$488,000	Lee et al. 2009 [41]

Lifetime value of a statistical life	EPA valuation	$7,400,000	NCEE, 2006^*∗*^
	Prime working age	$7,000,000	Viscusi and Aldy 2003 [42]

^*∗*^National Center for Environmental Economics (NCEE), http://yosemite.epa.gov/ee/epa/eed.nsf/pages/MortalityRiskValuation.html#howvalueVMR.
